# Supplementary material for: A model of human neural networks reveals NPTX2 pathology in ALS and FTLD
Source: Nature. 2024 Feb 14;626(8001):1073–83. doi: 10.1038/s41586-024-07042-7 (PMC10901740; doi:10.1038/s41586-024-07042-7)
Supplement: Supplementary file 1 — Supplementary Figs. 1–5 and Supplementary Tables 9–11. [file 41586_2024_7042_MOESM1_ESM.pdf]

nature portfolio

https://doi.org/10.1038/s41586-024-07042-7

Supplementary information

A model of human neural networks reveals NPTX2 pathology in ALS and FTLD

In the format provided by the authors and unedited

SI Guide to

A model of human neural networks reveals NPTX2 pathology in ALS/FTLD

Marian Hruska-Plochan[1], Vera I. Wiersma[1,13], Katharina M. Betz[1,2,3,13], Izaskun Mallona[1,2,3,13], Silvia Ronchi[4,12,13], Zuzanna Maniecka[1], Eva-Maria Hock[1], Elena Tantardini[1], Florent Laferriere[1], Sonu Sahadevan[1], Vanessa Hoop[2], Igor Delvendahl[2], Manuela Pérez-Berlanga[1], Beatrice Gatta[1], Martina Panatta[1], Alexander van der Bourg[5], Dasa Bohaciakova[6], Puneet Sharma[7,8], Laura De Vos[1], Karl Frontzek[9], Adriano Aguzzi[9], Tammaryn Lashley[10,11], Mark D. Robinson[2,3], Theofanis Karayannis[5], Martin Mueller[2], Andreas Hierlemann[4] & Magdalini Polymenidou[1#]

Table of contents

- **Supplementary Figure 1 Cluster markers and known marker genes of iCoMoNSCs** *(this PDF)*
  a, Heatmap showing top cluster markers of iCoMoNSCs clusters. **b,** Heatmap showing gene expression of a set of known markers amongst the iCoMoNSCs clusters. **c**, UMAP of iCoMoNSCs integrated with cells from three human NSC lines showing individual samples in different colors.

- **Supplementary Figure 2 Neuronal and glial maturation of iNets** *(this PDF)*
  a, UMAP of iCoMoNSCs and young, middle and old iNets (in replicates) showing cells from individual samples (different wells from the same differentiation) in different colors. **b**, Heatmap of cell distribution from all experimental stages amongst all clusters. **c**, Top marker genes for each cluster. **d**, UMAP with normalized expression of selected NSC, (**e**) glial, (**f**) pericyte and (**g**) neuronal marker genes across all samples. **h**, Heatmap with the expression of the top cluster markers from (**c**) from our aging experiment.

- **Supplementary Figure 3 Neuronal and glial maturation of iNets** *(this PDF)*
  Heatmap with the gene expression of known marker genes amongst all clusters from our aging experiment.

- **Supplementary Figure 4 and 5 Uncropped western blots** *(this PDF)*
  Compilation of all raw data obtained by electrophoretic separation.

- **Supplementary Table 1 DEG Cluster 12 vs other neuronal clusters upregulated (scRNA-seq)** *separate Excel file)*
  Genes identified by scRNA-seq as cluster 12 markers that are upregulated upon TDP-43-HA overexpression in iNets compared to other neuronal clusters.

- **Supplementary Table 2 DEG Cluster 12 vs other neuronal clusters downregulated (scRNA-seq)** *(separate Excel file)*
  Genes identified by scRNA-seq as cluster 12 markers that are downregulated upon TDP-43-HA overexpression in iNets compared to other neuronal clusters.

- **Supplementary Table 3 DEG Cluster 12 in TDP-43-negative FTLD-ALS patient nuclei** *(separate Excel file)*

Differential expression status of top up- and downregulated genes in cluster 12 in TDP-43-negative nuclei in FTLD-ALS patient brains.

- **Supplementary Table 4 DEG TDP-43-HA ON vs OFF (bulk)** *(separate Excel file)* Differentially expressed genes identified by bulk RNA-seq of iNets overexpressing TDP-43-HA (ON) compared to transduced iNets lacking expression induction (OFF).

- **Supplementary Table 5 DEG TDP-43 KD vs Ctrl shRNA (bulk)** *(separate Excel file)* Differentially expressed genes identified by bulk RNA-seq of iNets with shRNA-mediated TDP-43 KD compared to control shRNA-treated iNets.

- **Supplementary Table 6 Spliced exons** *(separate Excel file)*
  Analysis of spliced exon events in iNets with TDP-43 KD and TDP-43-HA overexpression.

- **Supplementary Table 7 Shared genes (OE, KD, patient brain) downregulated** *(separate Excel file)* Genes downregulated upon TDP-43-HA overexpression (ON) in iNets, shRNA-mediated TDP-43 KD in iNets and in TDP-43-negative neurons in FTLD-ALS patient brains.

- **Supplementary Table 8 Shared genes (OE, KD, patient brain) upregulated** *(separate Excel file)* Genes upregulated upon TDP-43-HA overexpression (ON) in iNets, shRNA-mediated TDP-43 KD in iNets and in TDP-43-negative neurons in FTLD-ALS patient brains.

- **Supplementary Table 9 Demographics of cases used in the present study** *(this PDF)* Overview of demographic data on human subjects of which post-mortem brain tissue was used in the present study

- **Supplementary Table 10 Antibody list** *(this PDF)*
  Overview of primary and secondary antibodies used in the present study.

- **Supplementary Table 11 Primer and shRNA hairpin sequences** *(this PDF)* PCR primers for Q5 polymerase site-directed mutagenesis cloning of shRNAs into MHP_shRNA cassette and resulting shRNA hairpin sequences.

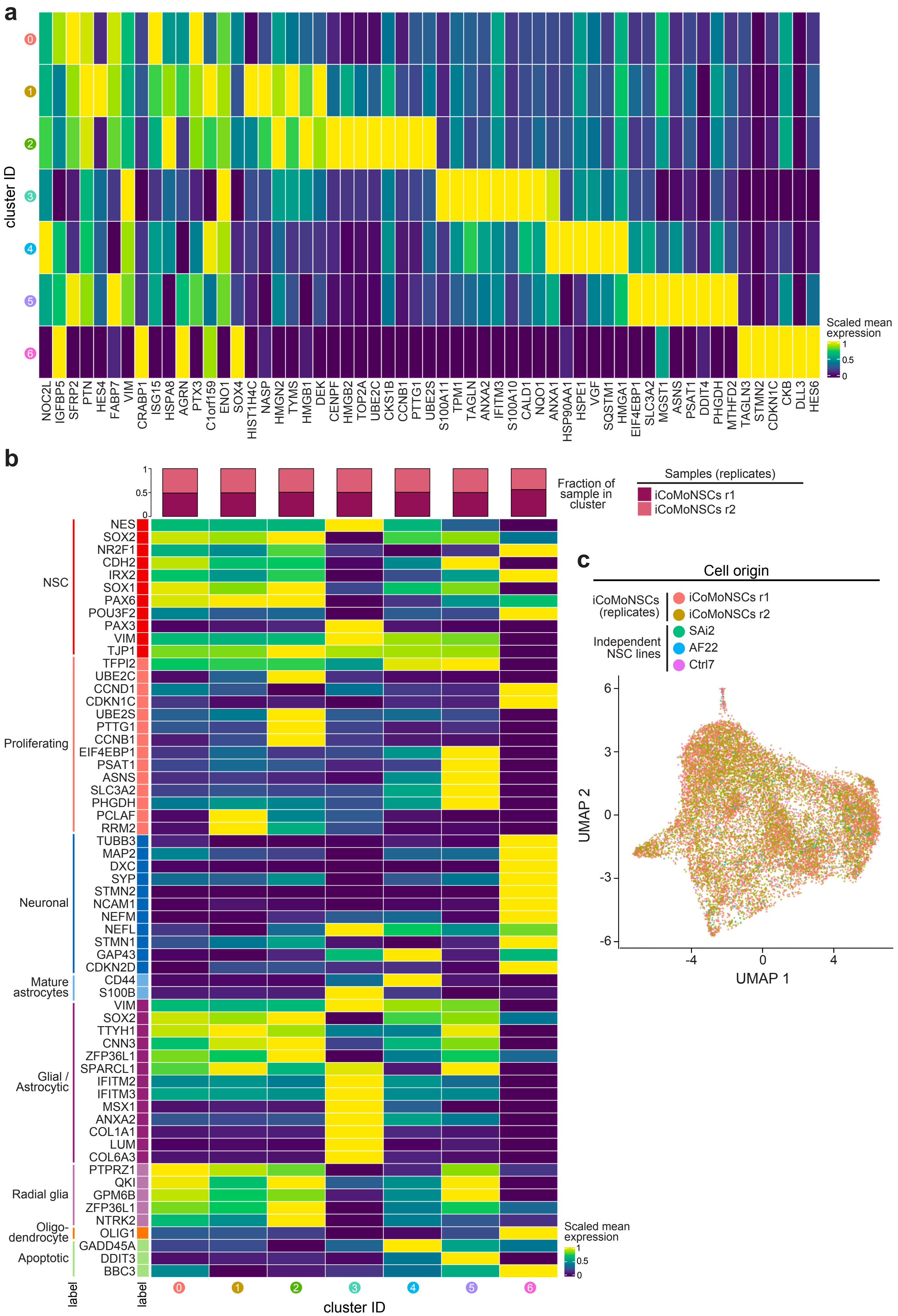

Supplementary Fig. 1

Supplementary Figure 1 Cluster markers and known marker genes of iCoMoNSCs. a, Heatmap showing top cluster markers of iCoMoNSCs clusters. **b,** Heatmap showing gene expression of a set of known markers amongst the iCoMoNSCs clusters. **c**, UMAP of iCoMoNSCs integrated with cells from three human NSC lines showing individual samples in different colors.

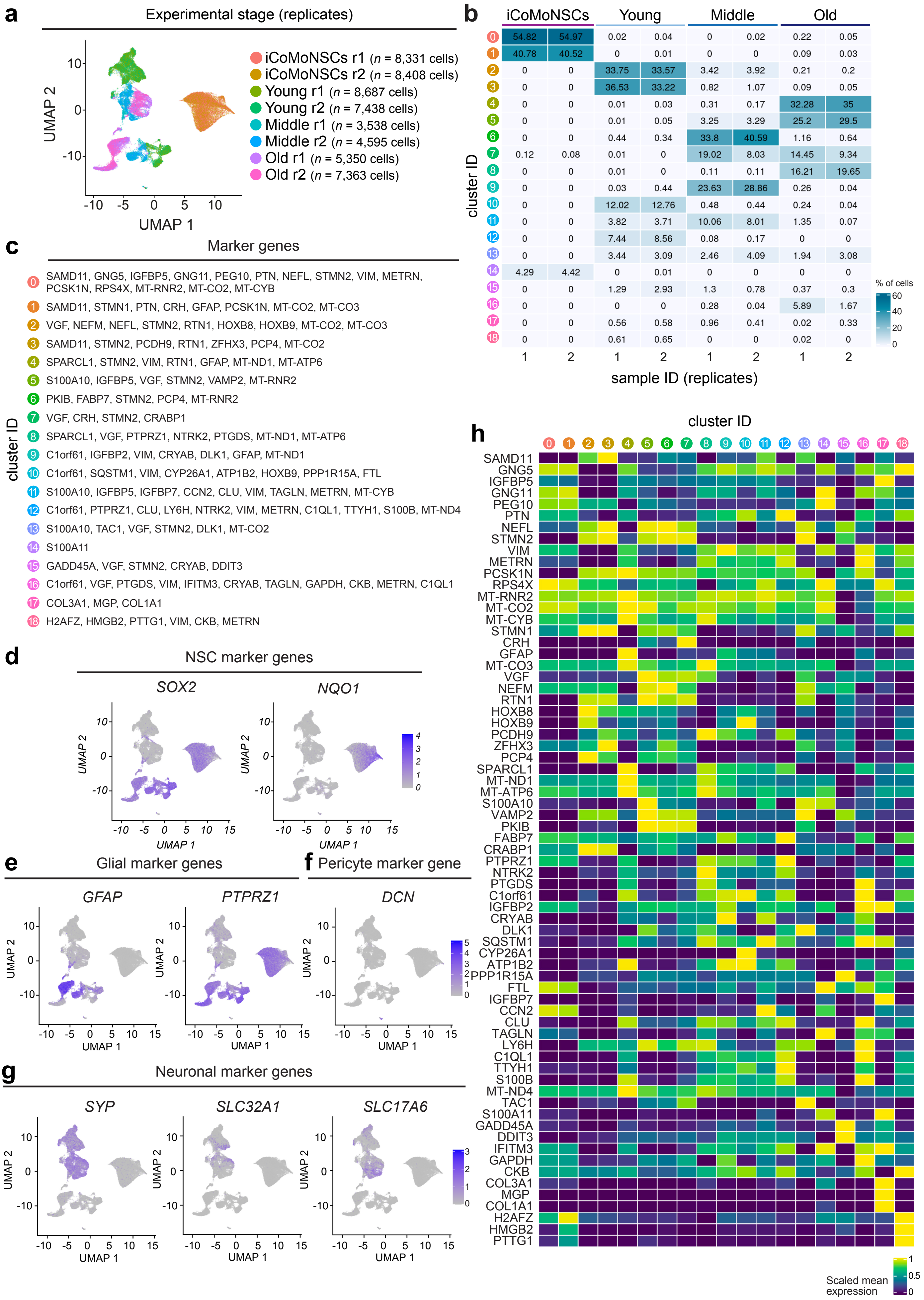

Supplementary Fig. 2

Supplementary Figure 2 Neuronal and glial maturation of iNets a, UMAP of iCoMoNSCs and young, middle and old iNets (in replicates) showing cells from individual samples (different wells from the same differentiation) in different colors. **b**, Heatmap of cell distribution from all experimental stages amongst all clusters. **c**, Top marker genes for each cluster. **d**, UMAP with normalized expression of selected NSC, (**e**) glial, (**f**) pericyte and (**g**) neuronal marker genes across all samples. **h**, Heatmap with the expression of the top cluster markers from (**c**) from our aging experiment.

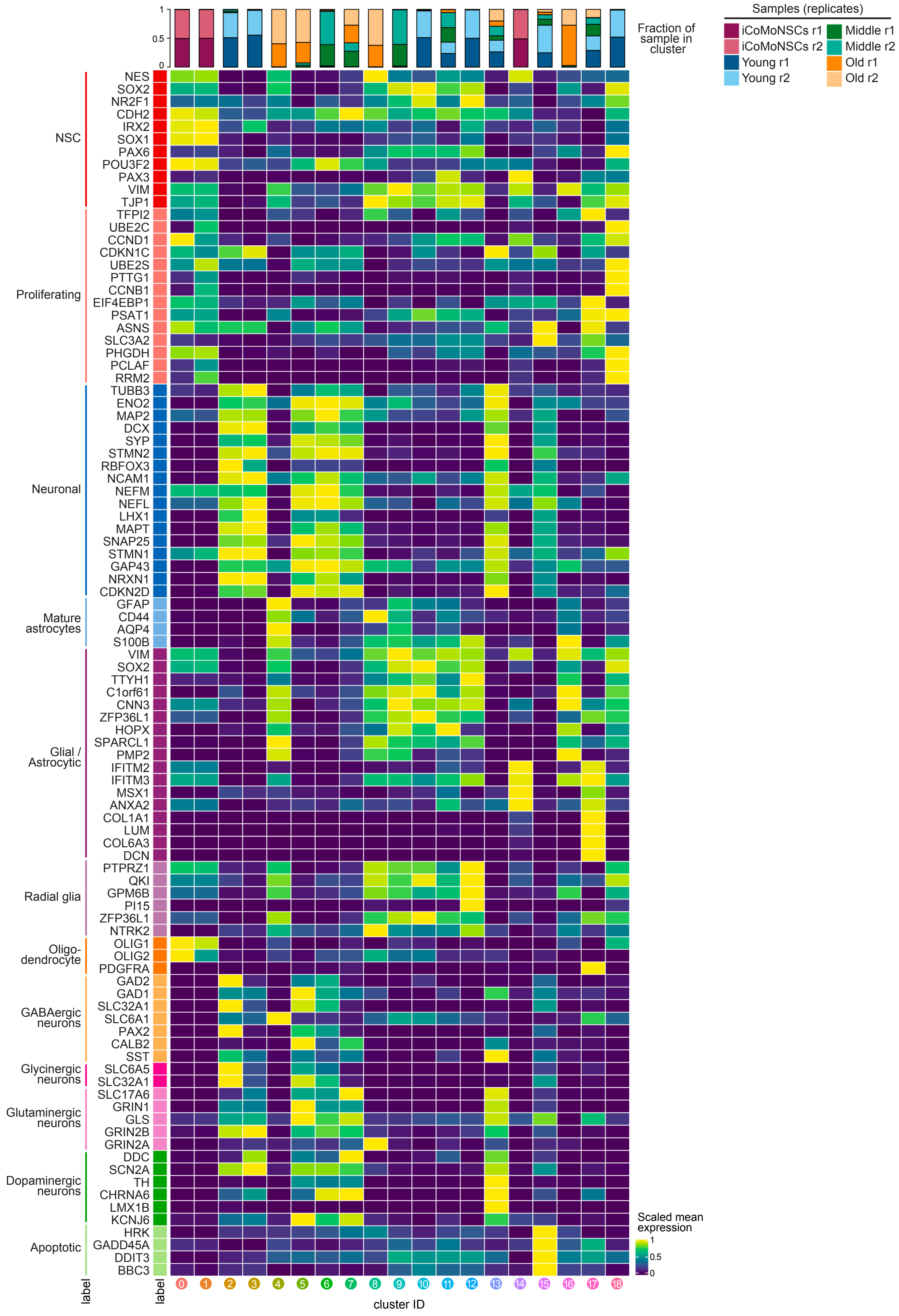

Supplementary Fig. 3

Supplementary Figure 3 Heatmap with the gene expression of known marker genes amongst all clusters from our aging experiment.

a Related to Figure 1j

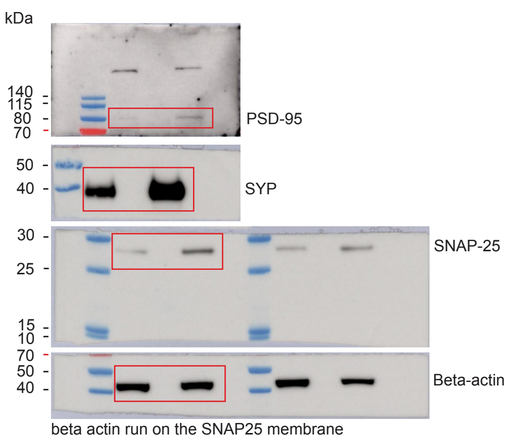

b Related to Figure 3c

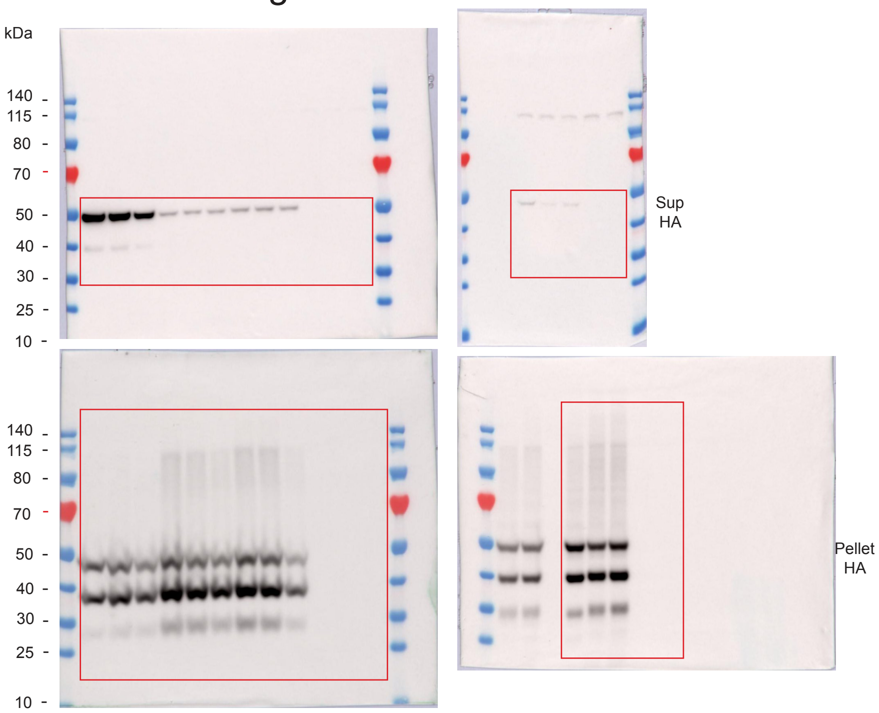

c Related to Extended data Figure 5 c and e

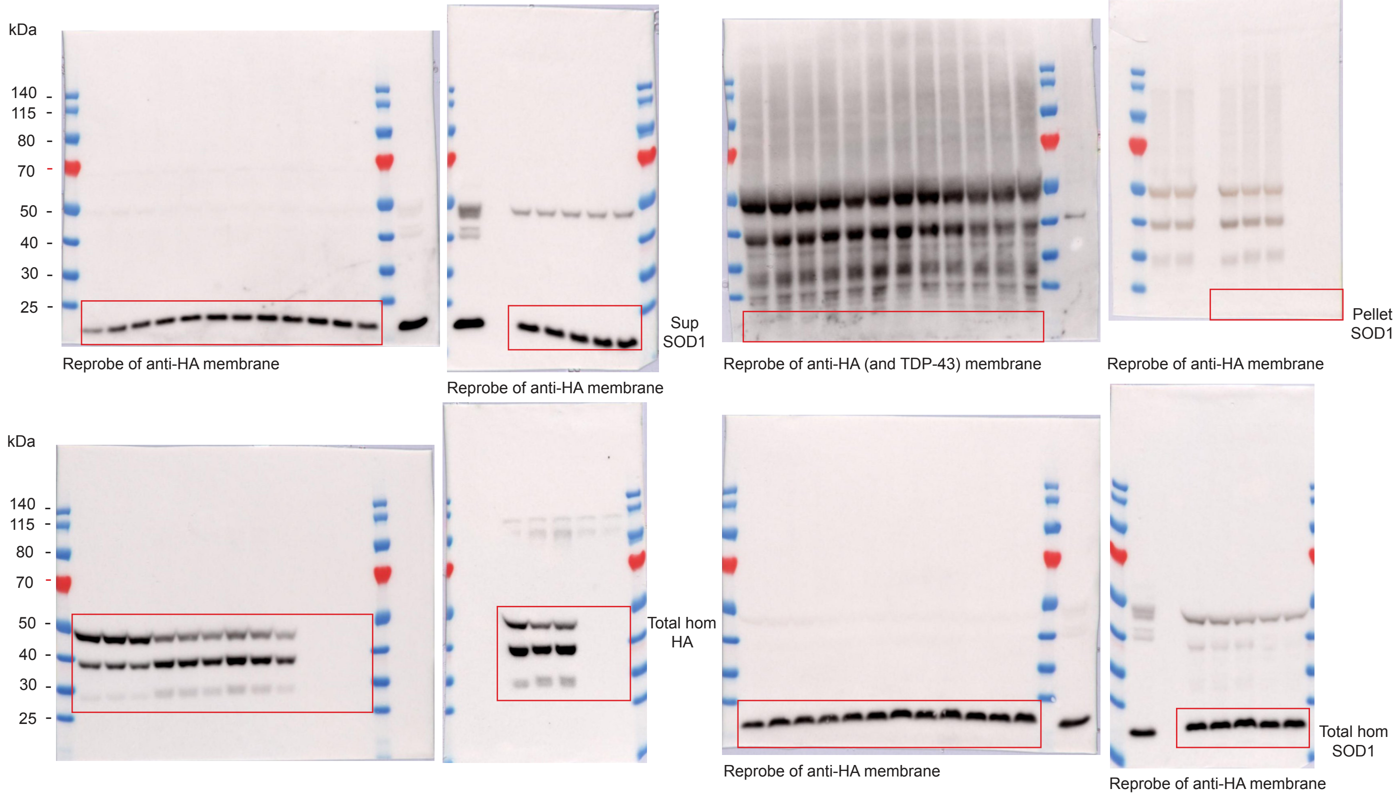

Supplementary Fig. 4

Supplementary Figure 4 Uncropped western blots. Compilation of raw data obtained by electrophoretic separation.

a Related to Extended data Figure 7 c

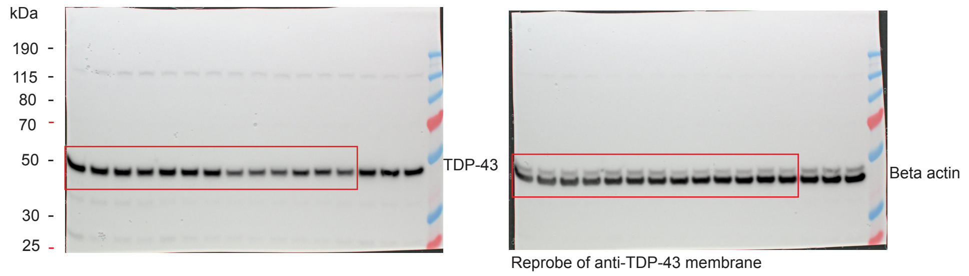

b Related to Extended data Figure 8 c

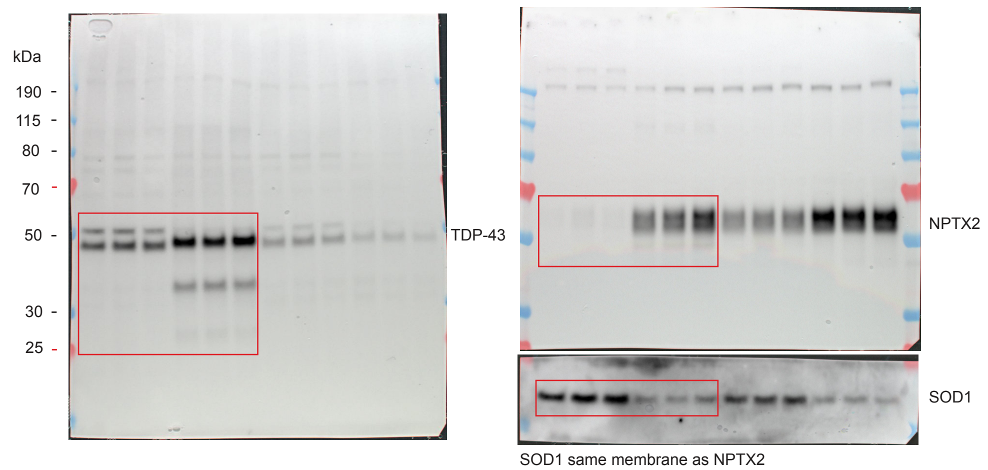

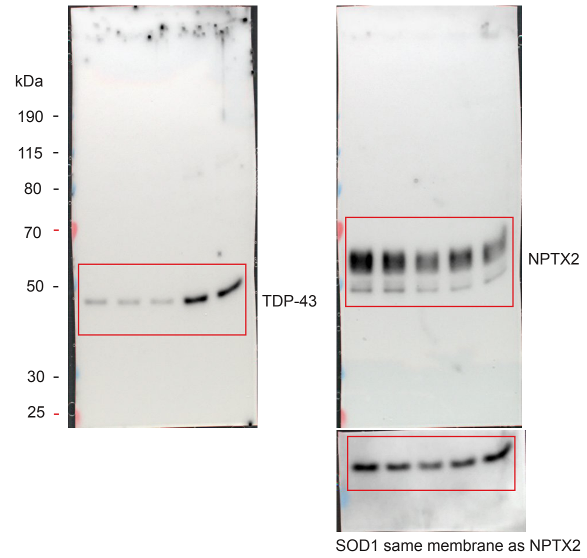

c Related to Extended data Figure 13 c

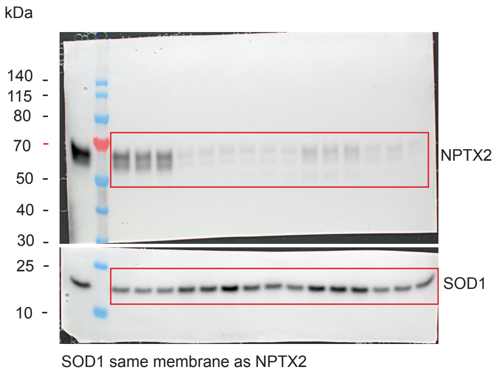

d Related to Extended data Figure 13 e

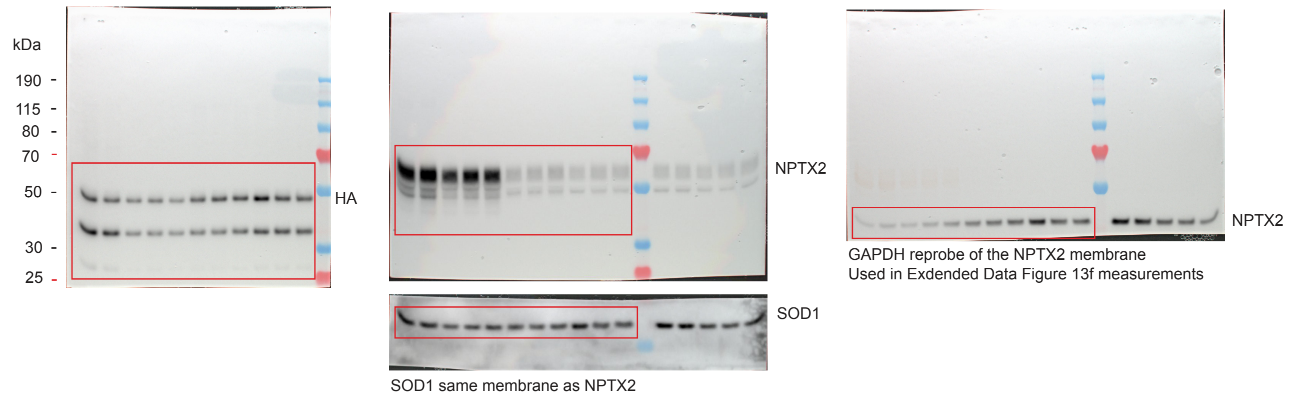

Supplementary Fig. 5

Supplementary Figure 5 Uncropped western blots. Compilation of raw data obtained by electrophoretic separation.

Supplementary Table 9 Demographics of cases used in the present study

Cases	Neuropathological diagnosis	Mutation	Gender	Age at disease onset	Age at death	Disease duration (years)	Post-mortem delay (h:min)
1	FTLD-TDP, Type A		F	57	63	6	85:20
2	FTLD-TDP, Type A	C9orf72	M	51	61	10	35:15
3	FTLD-TDP, Type A	C9orf72	F	56	67	11	85:35
4	FTLD-TDP, Type A		M	59	70	11	44:05
5	FTLD-TDP, Type C		M	64	74	10	19:00
6	FTLD-TDP, Type C		M	71	76	5	39:30
7	FTLD-TDP, Type C		F	58	73	15	37:55
8	ALS		F	62	62	0.58	46:00
9	FTLD-FUS		M	44	46	2	96:00
10	FTLD-FUS		M	49	59	10	81:35
11	FTLD-FUS		M	n.a.	67	n.a.	33:05
12	FTLD-Tau	MAPT 10+16	M	59	66	7	58:10
13	FTLD-Tau	MAPT 10+16	M	45	51	6	52:35
14	FTLD-Tau	MAPT L284R	M	41	45	4	27:55
15	FTLD-Tau	MAPT Q351R	F	36	69	33	82:55
16	AD		F	46	61	15	47:25
17	AD		M	54	65	11	34:25
18	AD		M	48	63	15	31:42
19	AD *		M	76	82	6	24:00
20	AD *		F	59	79	20	30:25

AD Alzheimer's disease, *ALS* amyotrophic lateral sclerosis, *C9orf72* Chromosome 9 open reading frame 72, *F* female, *FTLD-FUS* frontotemporal lobar degeneration with FUS proteinopathy, *FTLD-Tau* frontotemporal lobar degeneration with tauopathy, *FTLD-TDP* frontotemporal lobar degeneration with TDP-43 proteinopathy, *h:min* hours:minutes, *M* male, *MAPT* microtubule associated protein tau, *n.a.* not available, * TDP-43 co-pathology

Supplementary Table 10 Antibody list

Primary antibodies

Name	Species, Source	WB dilution	Cell IF dilution	Brain IF dilution
AQP4	Rb, Novus Biologicals #NBP1-87679	-	1:200	-
DCX	Gt, Santa Cruz Biotechnology #sc-8066 DCX	-	1:2000	-
FUS	Ms, ProteinTech #60160-1-Ig	-	-	1:50
GFAP	Gt, Abcam #ab53554	-	1:500	-
HA	Rb, Cell Signaling Technology #3724	1:2500	1:500	-
HA	Ms, Biolegend #901516	-	1:1000	-
HA	Ms, ThermoFisher #26183	-	1:500	-
KI67	Rb, Abcam #ab16667	-	1:250	-
MAP2	Ms, Sigma #M1406	-	1:250	-
MAP2	Ch, Abcam #ab5392	-	1:1000	1:1000
MEF2A	Rb, Santa Cruz Biotechnology #sc-17785	-	1:1000	-
NEFL	Ms, Thermo Scientific #13-0700	-	1:2000	-
Nestin	Ch, Online antibodies #ABIN187958	-	1:100	-
NEUN	Ch, Millipore #ABN91	-	1:1000	-
NPTX2	Rb, Proteintech #10889-1-AP	-	1:200	1:100
NUMA	Rb, Bethyl #A301-510A	-	1:200	-
PLZF	Rb, Santa Cruz Biotechnology #sc22839	-	1:200	-
PSD-95	Ms, Abcam #ab2723-100	1:2000	-	-
SNAP-25	Ms, #SMI81	1:1000	1:500	-
SOD1	Rb, Enzo #ADI-SOD-100	1:15000	-	-
SOX2	Gt, Santa Cruz Biotechnology #sc17320	-	1:250	-
STMN2	Ms, Proteintech #67204-1-Ig	-	1:100	-
STMN2	Rb, Proteintech #10586-1-AP	-	1:200	-

SYP	Rb, Santa Cruz Biotechnology #sc-9116	1:500	1:200	-
Tau $^{p202/205}$	Ms, ThermoFisher #MN1020	-	-	1:600
TDP-43$^{p403/404}$	Ms/Hu, custom-made	-	1:500	1:500
TDP-43$^{p403/404}$	Hu, custom-made	-	-	1:500
VIM	Ch, Millipore #AB5733	-	1:2000	-
ZO1	Rb, Millipore #AB2272	-	1:500	-
β-ACTIN	Ms, Sigma #A5441	1:5000	-	-
TDP-43 FL	Rb, Proteintech #18280-1-AP	1:1000	-	-
TDP-43 3H8	Ms, Novus #NBP1-92695	1:1000	-	-

Secondary antibodies

Name	Source	WB dilution	Cell IF dilution	Brain IF dilution
Donkey anti-Ch 488	Jackson Immuno Research #JAC703-546-155	-	1:500	-
Donkey anti-Ch 568	Jackson Immuno Research #JAC703-586-155	-	1:500	-
Donkey anti-Ch 647	Jackson Immuno Research #JAC703-606-155	-	1:500	-
Donkey anti-Gt 488	ThermoFisher #A11055	-	1:500	-
Donkey anti-Gt 594	ThermoFisher #A11058	-	1:500	-
Donkey anti-Gt 647	ThermoFisher #A21447	-	1:500	-
Donkey anti-Ms 488	ThermoFisher #A21202	-	1:500	-
Goat anti-Ms 555 PLUS	ThermoFisher #A48287	-	-	1:400
Donkey anti-Ms 568	ThermoFisher #A10037	-	1:500	1:400
Donkey anti-Ms 647	ThermoFisher #A31571	-	1:500	-
Donkey anti-Rb 488	ThermoFisher #A21206	-	1:500	1:400
Donkey anti-Rb 488 PLUS	ThermoFisher #A32790	-	-	1:400
Donkey anti-Rb 568	ThermoFisher #A10042	-	1:500	-
Donkey anti-Rb 647	ThermoFisher #A31573	-	1:500	-
Goat anti-Ch	ThermoFisher #A21449	-	1:500	1:400

647				
Goat anti-Ch 647 PLUS	ThermoFisher #A32933	-	-	1:400
Goat anti-Ms-HRP	Jackson Immuno Research #115-035-146	1:5000	-	-
Goat anti-Rb-HRP	Jackson Immuno Research #115-035-144	1:10000	-	-

Ch chicken, *Gt* goat, *HRP* horseradish peroxidase, *IF* immunofluorescence, *Ms* mouse, *Rb* rabbit, *WB* Western blot

Supplementary Table 11 Primer and shRNA hairpin sequences

**shNPTX2a_F primer**
5′-TGCTTAAAGGCGCTATTGCCTCTTTTTTTAATTAACATGGTCCCAGC-3′.
**shNPTX2a_R primer**
5′-AGCACAGCTTAAAGGCGCTATTGCCTCAAGCTTTCGTCCTTTCCAC-3′.
**shNPTX2a hairpin**
5′-GAGGCAATAGCGCCTTTAAGCTGTGCTTGCTTAAAGGCGCTATTGCCTCTT-3′.

**shNPTX2b_F primer**
5′-TGCTGGCCTCGCGCTGCGCGCCTTTTTTTAATTAACATGGTCCCAGC-3.
**shNPTX2b_R primer**
5′-AGCACAGCTGGCCTCGCGCTGCGCGCCAAGCTTTCGTCCTTTCCAC-3′.
**shNPTX2b hairpin**
5′-GGCGCGCAGCGCGAGGCCAGCTGTGCTTGCTGGCCTCGCGCTGCGCGCCTT-3′.

**shNPTX2c_F primer**
5′-TGCTAAATTACTACTCCCGTCCTTTTTTTAATTAACATGGTCCCAGC-3′.
**shNPTX2c_R primer**
5′-AGCACAGCTAAATTACTACTCCCGTCCAAGCTTTCGTCCTTTCCAC-3′.
**shNPTX2c hairpin**
5′-GGACGGGAGTAGTAATTTAGCTGTGCTTGCTAAATTACTACTCCCGTCCTT-3′.

**shNPTX2d_F primer**
5′-TGCTAATGCCATAGCTAGTGATTTTTTTTAATTAACATGGTCCCAGC-3′.
**shNPTX2d_R primer**
5′-AGCACAGCTAATGCCATAGCTAGTGATAAGCTTTCGTCCTTTCCAC-3′.
**shNPTX2d hairpin**
5′-ATCACTAGCTATGGCATTAGCTGTGCTTGCTAATGCCATAGCTAGTGATTT-3′.

**shTDP-43b_F primer**
5′-TTGCTTAGAATTAGGAAGTTTGCTTTTTTTAATTAACATGGTCCCAG-3′.
**shTDP-43b_R primer**
5′-GCACAGCTTAGAATTAGGAAGTTTGCAAGCTTTCGTCCTTTCCAC-3′.
**shTDP-43b hairpin**
5′-GCAAACTTCCTAATTCTAAGCTGTGCTTGCTTAGAATTAGGAAGTTTGCTT-3′.

**shTDP-43c_F primer**
5′-TTGCTAATGATCAAGTCCTCTCCTTTTTTTAATTAACATGGTCCCAG-3′.
**shTDP-43c_R primer**
5′-GCACAGCTAATGATCAAGTCCTCTCCAAGCTTTCGTCCTTTCCAC-3′.
**shTDP-43c hairpin**
5′-GGAGAGGACTTGATCATTAGCTGTGCTTGCTAATGATCAAGTCCTCTCCTT-3′.

**shHaloTag_F primer**
5′-TGCTAAATGCAATACCTTTGACTTTTTTTAATTAACATGGTCCCAGC-3′.
**shHaloTag_R primer**
5′-AGCACAGCTAAATGCAATACCTTTGACAAGCTTTCGTCCTTTCCAC-3′.
**shHaloTag hairpin**
5′-GTCAAAGGTATTGCATTTAGCTGTGCTTGCTAAATGCAATACCTTTGACTT-3′.

**shEGFP1_F primer**
5′-GCTAGACGTTGTGGCTGTTGTTTTTTTTAATTAACATGGTCCCAGC-3′.
**shEGFP1_R primer**
5′-AAGCACAGCTAGACGTTGTGGCTGTTGTAAGCTTTCGTCCTTTCCAC-3′.
**shEGFP1 hairpin**
5′-ACAACAGCCACAACGTCTAGCTGTGCTTGCTAGACGTTGTGGCTGTTGTTT-3′.

**shEGFP2_F primer**

5′-GCTGATATAGACGTTGTGGCTTTTTTTTAATTAACATGGTCCCAGC-3′.
**shEGFP2_R primer**
5′-AAGCACAGCTGATATAGACGTTGTGGCTAAGCTTTCGTCCTTTCCAC-3′.
**shEGFP2 hairpin**
5′-AGCCACAACGTCTATATCAGCTGTGCTTGCTGATATAGACGTTGTGGCTTT-3′.

**shHNRNPK_F primer**
5′-TGCTTAAGCATTCCACAGCATCTTTTTTTAATTAACATGGTCCCAGC-3′.
**shHNRNPK_R primer**
5′-AGCACAGCTTAAGCATTCCACAGCATCAAGCTTTCGTCCTTTCCAC-3′.
**shHNRNPK hairpin**
5′-GATGCTGTGGAATGCTTAAGCTGTGCTTGCTTAAGCATTCCACAGCATCTT-3′.

PCR primers for Q5 polymerase site-directed mutagenesis cloning of shRNAs into MHP_shRNA cassette and resulting shRNA hairpin sequences
