# Supplementary material for: A model of human neural networks reveals NPTX2 pathology in ALS and FTLD
Source: Nature. 2024 Feb 14;626(8001):1073–83. doi: 10.1038/s41586-024-07042-7 (PMC10901740; doi:10.1038/s41586-024-07042-7)
Supplement: Supplementary file 2 — Reporting Summary [file 41586_2024_7042_MOESM2_ESM.pdf]

nature portfolio

Corresponding author(s): Magdalini Polymenidou

Last updated by author(s): 2023 - 11 - 23

Reporting Summary

Nature Portfolio wishes to improve the reproducibility of the work that we publish. This form provides structure for consistency and transparency in reporting. For further information on Nature Portfolio policies, see our Editorial Policies and the Editorial Policy Checklist.

Statistics

For all statistical analyses, confirm that the following items are present in the figure legend, table legend, main text, or Methods section.

n/a	Confirmed	
☐	☒	The exact sample size (*n*) for each experimental group/condition, given as a discrete number and unit of measurement
☐	☒	A statement on whether measurements were taken from distinct samples or whether the same sample was measured repeatedly
☐	☒	The statistical test(s) used AND whether they are one- or two-sided<br>*Only common tests should be described solely by name; describe more complex techniques in the Methods section.*
☐	☒	A description of all covariates tested
☐	☒	A description of any assumptions or corrections, such as tests of normality and adjustment for multiple comparisons
☐	☒	A full description of the statistical parameters including central tendency (e.g. means) or other basic estimates (e.g. regression coefficient) AND variation (e.g. standard deviation) or associated estimates of uncertainty (e.g. confidence intervals)
☐	☒	For null hypothesis testing, the test statistic (e.g. *F*, *t*, *r*) with confidence intervals, effect sizes, degrees of freedom and *P* value noted<br>*Give P values as exact values whenever suitable.*
☒	☐	For Bayesian analysis, information on the choice of priors and Markov chain Monte Carlo settings
☒	☐	For hierarchical and complex designs, identification of the appropriate level for tests and full reporting of outcomes
☒	☐	Estimates of effect sizes (e.g. Cohen's *d*, Pearson's *r*), indicating how they were calculated

Our web collection on statistics for biologists contains articles on many of the points above.

Software and code

Policy information about availability of computer code

Data collection	Leica Application Suite X for microscopy. EVOS M7000 Imaging System Software for microscopy. MetaXpress (version 6.7.1) for microscopy. InCell Analyzer 2500 (version 7.1). ScanImage for microscopy. Amersham Imager 600RGB integrated software and Fusion FX6 EDGE imager integrated software for western blot imaging. HD-MEA data were recorded using MaxLab Live.
Data analysis	Images acquired on confocal microscopes were deconvoluted using SVI Huygens (version 22100p3) and processed for visualization in Fiji (20230118). Widefield and MetaXpress images were pixel segmented in Ilastik (version 1.3.3post3) and quantified in Fiji. Nuclei signal intensity data was analyzed in CellProfiler (version 4.2.5). Western blots were processed and analyzed in Fiji. Lentiviral vector titers were checked using Takara GoStix Plus app (version 3.0.2). ImageJ (release 1.54g) and Matlab (version 2016a) were used to analyze 2-photon calcium imaging data. Patch clamp data were analyzed in Igor Pro. Data analysis of HD-MEA data was performed using custom-written codes in MATLAB (R2021a) and (Python 3.6.10) and Kilosort2 software within the SpikeInterface framework for spike sorting. Statistical analysis was performed in Prism (version 9.5.0) unless otherwise mentioned.<br><br>Data was processed with CellRanger for demultiplexing, read alignment to the human reference genome (GRCh38) and filtering to generate a feature-barcode matrix per sample. Cell doublets were removed with scDblFinder64 and outlier cells were detected and filtered with the “scater” R package. Seurat v3 was used for log-normalization and to identify the top 2000 highly variable genes per sample. Marker genes that are upregulated in one cluster compared to any other cluster were identified with the findMarkers function from the scran R package. scRNAseq samples were integrated in Seurat v3. Batch effects were assessed with the smoothed `cms` mixing metric from CellMixS. CellRanger was used for quantification of the expression of the TDP-43-HA construct components. The total TDP-43 log2FC between cluster 12 and all other neuronal clusters was computed using the `summary.logFC` metric from scran’s findMarkers. Pseudotime analysis was carried out with monocle3 v1.0.0, commit 004c096, rooting the trajectories in our annotated NSC cluster. MetaNeighbor v1.8.0 to measure cell-type replication across experiments using the annotated clusters. Raw reads from bulk RNA seq were processed with ARMOR76. Briefly, reads were

aligned and counted to the human genome (GRCh38 assembly and Gencode release 43) with salmon v1.4.0 and with STAR 2.7.7a. We modeled the salmon-generated count data with quasi-likelihood (QL) negative binomial (NB) generalized log-linear models and ran differential expression analysis with edgeR v3.36.0. We reanalyzed CLiP data from GSE27201 Polymenidou 2011 (mouse) and Tollervey 2011 (human) with the nf-core/clipseq workflow v1.0.077 with default parameters and against the GRCh38 or GRCm38 reference genomes, as appropriate. Polymenidou 2011 CLiP binding sites were transformed from GRCm38 to GRCh38 coordinates using liftOver from Kent utils v39078. CLiP and iCLiP data was re-analysed using the GRCh38 reference genome with the nf-core/clipseq77 for preprocessing, mapping and crosslink site identification. Re-analysis of RNA-seq data from sorted nuclei was performed via differential gene expression analysis between TDP-43 negative and positive nuclei was performed in R version 4.0.5 with edgeR taking into account the paired nature of the data using the quasi-likelihood framework.

Splicing analysis to identify skipped exons was performed using the human reference genome (GRCh38) and annotation (version 108) were obtained from ENSEMBL91. Illumina Trueseq adaptors were removed from RNA-seq reads using cutadapt (version 4.1) with the parameters -q 25 -m 25 . The processed reads were mapped to the reference genome using STAR aligner (version 2.7.10b) with default parameters. Subsequently, differential splicing analysis at the event level was performed using rMATS (version 4.1.2) with the parameters -t paired --readLength 100 --variable-read-length. Significant skipped exons events were defined by FDR < 0.05 and 15 % change in absolute value of IncLevelDifference and intersected with a human skipped exon dataset43. The results were processed using tidyverse package (v2.0.0) in R. Upset plot was prepared using Complexheatmap package (v2.17.0) in R.

Code is deposited on Zenodo with doi https://doi.org/10.5281/zenodo.8142336.

For manuscripts utilizing custom algorithms or software that are central to the research but not yet described in published literature, software must be made available to editors and reviewers. We strongly encourage code deposition in a community repository (e.g. GitHub). See the Nature Portfolio guidelines for submitting code & software for further information.

Data

Policy information about availability of data

All manuscripts must include a data availability statement. This statement should provide the following information, where applicable:

- Accession codes, unique identifiers, or web links for publicly available datasets
- A description of any restrictions on data availability
- For clinical datasets or third party data, please ensure that the statement adheres to our policy

Data availability
scRNA-seq and bulk RNA-seq data are available via GEO accession number GSE230647.

Other databases used in this study:
- The scRNA-seq data from Lam 2019 were downloaded from ArrayExpress (accession number E-MTAB-8379).
- Reanalyzed CLiP data from GSE27201 Polymenidou 2011 (mouse) including accessions SRR107031 and SRR107032; from E-MTAB-530 Tollervey 2011 (human) accessions ERR039847, ERR039848, ERR039843, ERR039842, ERR039844, ERR039845, ERR039846.
- FASTQ files of TDP-43 human brain iCLIP13 (three controls and three FTLD patients) were downloaded from ArrayExpress (E-MTAB-530).
- FASTQ files of TDP-43 CLiP in mouse were retrieved from GSE27201.
- We downloaded the RNA-seq gene count table as a supplementary data file (GSE126542_NeuronalNuclei_RNAseq_counts.txt.gz) from GEO accession GSE126542.

Code availability
Code is deposited on Zenodo with doi https://doi.org/10.5281/zenodo.8142336.

Research involving human participants, their data, or biological material

Policy information about studies with human participants or human data. See also policy information about sex, gender (identity/presentation), and sexual orientation and race, ethnicity and racism.

Reporting on sex and gender	Used sex to identify patient sex.
Reporting on race, ethnicity, or other socially relevant groupings	We do not report on race, ethnicity, or other socially relevant groups.
Population characteristics	Basic demographic information was provided with the human brain tissue, which was selected based on the underlying pathological diagnosis and the known mutations that were determined in the clinical setting. Other demographic data included age at onset, disease duration, age at death and sex. No other information was provided or was relevant for the present study.
Recruitment	The participants were recruited through the brain donation programme at Queen Square Brain Bank, therefore there is no self-selection biases. Cases identified for this project were selected based on their underlying pathological diagnosis.
Ethics oversight	Formalin-fixed, paraffin-embedded hippocampal, frontal or primary motor cortex patient (ALS, FTLD-TDP Type A, FTLD-TDP Type C, FTLD-FUS, FTLD-Tau, AD) sections (see Supplementary Table 9) were used. All FTLD and AD tissue samples were donated to Queen Square Brain Bank for Neurological Disorders at UCL Queen Square Institute of Neurology with full, informed consent and the material was supplied with an approved material transfer agreement (EXT MTA 07-2017). Anonymized autopsy ALS sample was collected by the Institute of Neuropathology at UZH. According to Swiss law, anonymized autopsy tissues do not fall within the scope of the Human Research Act and may be used in research.

Note that full information on the approval of the study protocol must also be provided in the manuscript.

Field-specific reporting

Please select the one below that is the best fit for your research. If you are not sure, read the appropriate sections before making your selection.

☒ Life sciences ☐ Behavioural & social sciences ☐ Ecological, evolutionary & environmental sciences

For a reference copy of the document with all sections, see nature.com/documents/nr-reporting-summary-flat.pdf

Life sciences study design

All studies must disclose on these points even when the disclosure is negative.

Sample size	For experiments with iNets, minimum of 3 individually treated wells (i.e. individually transduced) of imaging multi well plates (or a larger vessels for bulk RNA seq or biochemistry) were used per condition per experiment as this was previously determined to be sufficient sample size (Emmenegger et al. 2021 EMBO Molecular Medicine).<br><br>Luminescence experiments with HEK293T cells were also performed with at least 3 replicates (i.e. individually transfected and transduced wells). No size estimation was performed as the sufficient sample size was determined by trial experiments.<br><br>Minimum of 3 wells of 6 well plates per LV (shRNA) were used for SH-SY5Y LV transduction to determine shRNA efficacies. No size estimation was performed as the sufficient sample size was determined by previous experiments independent from this paper (Avar et al. 2022 The EMBO Journal).<br><br>4 wells of an ibidi chamber slide per LV were used in the primary mouse neuron experiment. No size estimation was performed as the sufficient sample size was determined by previous experiments independent from this paper .<br><br>Formalin-fixed, paraffin-embedded hippocampal, frontal or primary motor cortex patient (ALS, FTLD-TDP Type A, FTLD-TDP Type C, FTLD-FUS, FTLD-Tau, AD) sections (see Supplementary Table 9) were used based on availability, and at least 2 slides per patient per staining were used.
Data exclusions	No data were excluded.
Replication	All repeated experiments were successful (i.e. significant or same trend; did not fail; did not result in an opposite effect) and this is mentioned in the text or visualized in the figure legend.
Randomization	For all experiments with iNets and HEK293T cells, wells for different conditions were randomly selected (but kept within the rows or columns). SH-SY5Y cells for shRNA testing were randomly transduced in 6 well plates (but 2 lentiviral vectors were used per plate). Patient brain cases identified for this project were selected based on their underlying pathological diagnosis and availability.
Blinding	All iNets imaging quantification experiments were imaged with high-content microscopes and data were analyzed in one batch, i.e. the information about the sample type was only revealed and used when plotting the results of quantification and therefore blinding was not relevant.<br>For all other experiments, blinding was not possible due to nature of the experiments.

Reporting for specific materials, systems and methods

We require information from authors about some types of materials, experimental systems and methods used in many studies. Here, indicate whether each material, system or method listed is relevant to your study. If you are not sure if a list item applies to your research, read the appropriate section before selecting a response.

Materials & experimental systems

n/a	Involved in the study	
☐	☒	Antibodies
☐	☒	Eukaryotic cell lines
☒	☐	Palaeontology and archaeology
☐	☒	Animals and other organisms
☒	☐	Clinical data
☒	☐	Dual use research of concern
☒	☐	Plants

Methods

n/a	Involved in the study	
☒	☐	ChIP-seq
☒	☐	Flow cytometry
☒	☐	MRI-based neuroimaging

Antibodies

Antibodies used	Precise information about antibodies can be found in the Supplementary Table 10

Primary antibodies:
AQP4, Rb, Novus Biologicals #NBP1-87679
DCX, Gt, Santa Cruz Biotechnology #sc-8066 LOT E0115, clone C-18
FUS, Ms, ProteinTech #60160-1-Ig, LOT 10003284, clone 3A10B5
GFAP, Gt, Abcam #ab53554
HA, Rb, Cell Signaling Technology #3724, LOT 11, clone C29F4
HA, Ms, Biolegend #901516, LOT B324182, clone 16B12
HA, Ms, ThermoFisher #26183, LOT YB362804, clone 2-2.2.14
KI67, Rb, Abcam #ab16667, clone SP6
MAP2, Ms, Sigma #M1406, clone AP-20
MAP2, Ch, Abcam #ab5392, LOTs GR3426750-3, GR3450786-1
MEF2A, Rb, Santa Cruz Biotechnology #sc-17785, LOT D29121, clone B-4
NEFM, Ms, Thermo Scientific #13-0700, LOT RL246769, clone RMO-270
Nestin, Ch, Online antibodies #ABIN187958
NEUN, Ch, Millipore #ABN91, LOT 2695293
NPTX2, Rb, Proteintech #10889-1-AP, LOTs 00052993, 00053480
NUMA, Rb, Bethyl #A301-510A, LOT 1
PLZF, Rb, Santa Cruz Biotechnology #sc22839, LOT G1414, clone H300
PSD-95, Ms, Abcam #ab2723-100, LOT XE341719, clone 6G6-1C9
SNAP-25, Ms, #SMI81, clone SMI-81
SOD1, Rb, Enzo #ADI-SOD-100, LOTs 09082051, 03122119
SOX2, Gt, Santa Cruz Biotechnology #sc17320, LOT E0715, clone Y-17
STMN2, Ms, Proteintech #67204-1-Ig, LOT 10011368, clone 1F6C4
STMN2, Rb, Proteintech #10586-1-AP, LOT 00058414
SYP, Rb, Santa Cruz Biotechnology #sc-9116, clone H93
Tau p202/205, Ms, ThermoFisher #MN1020, LOT VL3113305, clone AT8
TDP-43p403/404, Custom made, Murinised human monoclonal
VIM, Ch, Millipore #AB5733, LOT 3822323
ZO1, Rb, Millipore #AB2272, LOT 2549491
β-ACTIN, Ms, Sigma #A5441, LOT 000126949, clone AC-15
TDP-43 FL, Rb, Proteintech #18280-1-AP, LOTs 00072915, 00025213
TDP-43 3H8, Ms, Novus #NBP1-92695, LOTs 022221, 082119, clone 3H8

Secondary antibodies:
Donkey anti-Ch 488, Jackson Immuno Research #JAC703-546-155, LOT 2420700
Donkey anti-Ch 568, Jackson Immuno Research #JAC703-586-155, LOT 141724
Donkey anti-Ch 647, Jackson Immuno Research #JAC703-606-155, LOT 157044
Donkey anti-Gt 488, ThermoFisher #A11055, LOT 1771339
Donkey anti-Gt 594, ThermoFisher #A11058, LOT 1445994
Donkey anti-Gt 647, ThermoFisher #A21447, LOT 1739289
Donkey anti-Ms 488, ThermoFisher #A21202, LOT 2428531
Goat anti-Ms 555 PLUS, ThermoFisher #A4287, LOT WH334377
Donkey anti-Ms 568, ThermoFisher #A10037, LOT 2300930
Donkey anti-Ms 647, ThermoFisher #A31571, LOT 2555690
Donkey anti-Rb 488, ThermoFisher #A21206, LOT 2289872
Donkey anti-Rb 488 PLUS, ThermoFisher #A32790, LOT VJ314112
Donkey anti-Rb 568, ThermoFisher #A10042, LOT 2540901
Donkey anti-Rb 647, ThermoFisher #A31573, LOT 2359136
Goat anti-Ch 647, ThermoFisher #A21449, LOT 1744743
Goat anti-Ch 647 PLUS, ThermoFisher #A32933, LOT XD344361
Goat anti-Ms-HRP, Jackson Immuno Research #115-035-146, LOT 165273
Goat anti-Rb-HRP, Jackson Immuno Research #115-035-144 LOT 163676

Validation

Precise information about antibodies can be found in the Supplementary Table 10

Primary antibodies:
AQP4, PMID:36803838, AB_11006038
DCX, PMID:26337870; PMID:2770949, AB_2088494
FUS, PMID:36171642, AB_10666169
GFAP, PMDI:36384142; PMID:35431801, AB_880202
HA, PMID:25380328, AB_1549585
HA, PMID:32515353, AB_2820200
HA, PMID:12119359, AB_10978021
KI67, PMID:24424056, AB_302459
MAP2, PMID:19058188, AB_477171
MAP2, PMID: 28521134; PMID: 28637841, AB_2138153
MEF2A, PMID: 29518074; PMID: 36263180, AB_627921
NEFM, PMID:28472866, AB_2532998
Nestin, PMID:32778115
NEUN, PMID:28555077, AB_11205760
NPTX2, PMID:29844474; PMID:34565284, AB_2153875
NUMA, AB_999641
PLZF, PMID:30759202, AB_2304760
PSD-95, PMID:30638745, AB_303248
SNAP-25, PMID:18041776, AB_2315336
SOD1, PMID:30044993, AB_10616253

SOX2, PMID:31883789, AB_2286684
STMN2, AB_2882497
STMN2, AB_2197283
SYP, PMID:29798891, AB_2199007
Tau p202/205, PMID:31325178, AB_223647
TDP-43p403/404, PMID:34806807
VIM, PMID:31461644, AB_11212377
ZO1, AB_10807434
β-ACTIN, PMID:31549732, AB_476744
TDP-43 FL, PMID:36303452, AB_2240312
TDP-43 3H8, PMID:37182104, AB_11005586

Eukaryotic cell lines

Policy information about cell lines and Sex and Gender in Research

Cell line source(s)	HEK293T (ATCC #CRL-3216) - collaborator Urs Greber (UZH). SH-SY5Y - Sigma (#94030304). Human early neonatal dermal fibroblasts - Gibco #C0045C (male).
Authentication	HEK293T cells used for luminescence assay and lentiviral production were provided by a collaborator (Urs Greber) and were authenticated by the vendor (ATCC #CRL-3216). SH-SY5Y were obtained directly from Sigma (#94030304) and thus authenticated by the vendor. iCoMoNSCs were generated in this manuscript from in-house generated iPSCs that were generated from human early neonatal dermal fibroblasts purchased directly from Invitrogen (Gibco #C0045C). Fibroblasts, iPSCs and iCoMoNSCs were checked for karyotype.
Mycoplasma contamination	All cycling cells used in the study were routinely checked for possible mycoplasma contamination using EZ PCR Mycoplasma Detection Kit (Sartorius #20-700-20). Young iNets (with some residual KI67+ cells) were sporadically checked, too. No mycoplasma contamination was detected.
Commonly misidentified lines (See ICLAC register)	Cell lines (SH-SY5Y Sigma #94030304 and HEK293T ATCC #CRL-3216) were checked against cross-contaminated or misidentified cell lines (https://iclac.org/databases/cross-contaminations/). No cross-contaminated or misidentified cell lines were identified.

Animals and other research organisms

Policy information about studies involving animals; ARRIVE guidelines recommended for reporting animal research, and Sex and Gender in Research

Laboratory animals	Mus musculus C57BL/6 strain. Mouse embryos (E16/17) were used.
Wild animals	No wild animals were used in the study.
Reporting on sex	Primary neurons were derived from hippocampi of all fetuses available - i.e. the resulting culture was made of mixed sex.
Field-collected samples	No field-collected samples were used.
Ethics oversight	Primary neuronal cell cultures were prepared from mouse embryos (E16/17). Pregnant C57BL/6 females were delivered from Janvier Labs (France) at day 12 of pregnancy. All animal experimentation, including mouse housing and breeding was done in accordance with the Swiss Animal Welfare Law and in compliance with the regulations of the Cantonal Veterinary Office, Zurich (current approved license ZH169/2022, valid until 16.02.2026).

Note that full information on the approval of the study protocol must also be provided in the manuscript.

Plants

Seed stocks	N/A
Novel plant genotypes	N/A
Authentication	N/A
